# Effect of a full moon on mortality of patients admitted to the ICU

**DOI:** 10.1186/cc11106

**Published:** 2012-03-20

**Authors:** R Nadeem, A Nadeem, E Madbouly, J Molnar, J Morrison

**Affiliations:** 1 Captain James A. Lovell Federal Healthcare Center, North Chicago, IL, USA; 2 Rosalind Franklin University of Medicine and Science, North Chicago, IL, USA

## Introduction

The effect of the full moon (the lunar effect) on human behaviour has occupied researchers for centuries. We aim to determine such a lunar effect on mortality among patients admitted to the ICU.

## Methods

We analyze the electronic medical records of patients admitted to the ICU. The subjects were divided into two groups: patients who died on full moon days (14th,15th, and 16th days of the lunar month) and the patients who died on other days of the lunar month. The mortality rates were calculated for patients in both groups. Parameters including age, gender, acute physiology and chronic health evaluation (APACHE) III scores, predicted mortality, type of ICU, and actual mortality were compared between the two groups. Student's *t *test was performed to determine whether there were any differences between the groups.

## Results

Data from 4,387 patients who were followed for 23 months were analyzed. Overall, 297 patients died during this period, including 31 patients on full moon days and 266 patients on the other days of the month. Both groups were similar in terms of age (73 vs. 71 years, *P *= 0.39), APACHE III scores (82.06 vs. 76.52, *P *= 0.28), and predicted mortality (0.405 vs. 0.370, *P *= 0.48). There was no difference in the frequency of death between the full moon days and the other days (10.33 vs. 9.85, *P *= 0.81). See Table 1.

**Table 1:** Characteristics of patients who died on full moon days versus other days

	Full moon	Other days	*P *value
Age	73.6 ± 14.59	71.07 ± 16.1	0.39
Male/female	15/16	133/133	0.86
APACHE III	82.06 ± 24.1	76.52 ± 27.4	0.28
Mortality	0.405 ± 0.249	0.370 ± 0.268	0.48

## Conclusion

The full moon does not seem to affect the mortality of patients admitted to the ICU.
